# Analysis of concordance with antiemetic guidelines in pediatric, adolescent, and young adult patients with cancer using a large‐scale administrative database

**DOI:** 10.1002/cam4.2486

**Published:** 2019-08-30

**Authors:** Seiko Bun, Susumu Kunisawa, Noriko Sasaki, Kiyohide Fushimi, Kimikazu Matsumoto, Akimasa Yamatani, Yuichi Imanaka

**Affiliations:** ^1^ Department of Healthcare Economics and Quality Management Graduate School of Medicine Kyoto University Kyoto Japan; ^2^ Department of Pharmacy National Center for Child Health and Development Hospital Setagaya‐ku Japan; ^3^ Department of Health Policy and Informatics Graduate School of Medicine Tokyo Medical and Dental University Bunkyo‐ku Japan; ^4^ Department of Children's Oncology Center National Center for Child health and Development Hospital Setagaya‐ku Japan

**Keywords:** adherence, administrative database, adolescent and young adult, antiemetic guideline, pediatrics

## Abstract

**Object:**

The appropriate use of antiemetics is important for the prevention of chemotherapy‐induced nausea and vomiting (CINV); however, little is known about the rate of concordance with antiemetic guidelines for CINV in the field of pediatric, adolescent, and young adult.

**Methods:**

Using the Diagnosis Procedure Combination system in Japan, we identified patients <30 years of age who were diagnosed with cancer between July 2010 and March 2016. We have assessed concordance with the ASCO antiemetic guidelines for each emetic risk category of chemotherapeutic drugs. Furthermore, we have assessed the risk factors of discordance with the antiemetic guidelines using a logistic regression.

**Results:**

In total, 21 106 patients who underwent chemotherapy were included. The rates of concordance with the guidelines in each emetic risk category of chemotherapeutic drugs were 51.1% in high risk, ≥18 years of age; 21.5% in high risk, <18 years of age; 32.1% in moderate risk; 52.0% in low risk; and 51.6% in minimal risk. The main reason for the discordance was underuse of antiemetics, especially steroids. The factors for discordance were younger age, use of moderate and high emetic risk chemotherapeutic drugs, hematological malignancy, and brain tumor.

**Conclusion:**

There is substantial scope to improve the antiemetic practice and reduce the risk of discordance with the antiemetic guidelines in pediatric, adolescent, and young adult patients. The risk factors are different from those in adults. Further investigations to evaluate the causes of discordance are warranted.

## Graphical abstract

This is the first large‐scale report of adherence to antiemetic guidelines for pediatrics, adolescents, and young adult patients who have received chemotherapy. Our study demonstrated that a concordance with the antiemetic guidelines in the pediatric population is lower than that in adults.

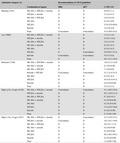

## 1 INTRODUCTION

Chemotherapy‐induced nausea and vomiting (CINV) is one of the most serious concerns for children and adolescent and young adult patients (AYA) with cancer,^1^ and is a leading cause of discontinuation of chemotherapy and it reduces the quality of life of patients. Over the past decade, clinical studies in the adult population have enabled the patients to receive newer drugs, such as 5‐hydroxytryptamine_3_ receptor antagonists (5HT_3_RA)^2^ or neurokinin‐1 receptor antagonists (NK‐1RA)^3^ for CINV.

The guidelines published from different societies, such as the American Society of Clinical Oncology (ASCO),^4^, ^5^ European Society for Medical Oncology (ESMO),^6^ and Japan Society of Clinical Oncology (JSCO),^7^ recommend the use of appropriate antiemetic drugs for patients receiving chemotherapy. The emetic risk of the chemotherapeutic drugs can be classified into four categories.^5^ The chemotherapeutic drugs in the absence of antiemetic prophylaxis that cause >90%, 30%‐90%, 10%‐30%, and <10% emesis are considered to be high, moderate, low, and minimal emetic risk, respectively.

Several studies conducted in the adult population have demonstrated concordance with the antiemetic guidelines, reporting a concordance rate of 70%‐90% in Japan^8^ and 70% in the European countries.^9^ The factors of discordance with the antiemetic guidelines in the adults are hematological malignancy, older age, and the use of low emetic risk chemotherapy.

On the other hand, in the pediatric patients, the Children's Oncology Group (COG), the world's largest clinical trials consortium have expressed concerns regarding discordance with the antiemetic guidelines.^10^ The possible factors are unfamiliarity of the medical personnel with the existing evidence and guidelines, drug‐drug interactions, and risk category of each chemotherapeutic drugs.

The scenario is, however, obscure in Japan. Hence, we have aimed to assess the concordance with the antiemetic guidelines in the pediatrics fields, together with adolescent, and young adult patients who are usually seen in the department of pediatrics department in Japan.

## 2 MATERIALS AND METHODS

### 2.1 Source of data

This was a retrospective observational study using the Diagnosis Procedure Combination (DPC) database, an administrative database in Japan. The data were obtained from approximately 80% of all the DPC hospitals which encompassed approximately 8 million inpatient admissions per year.^11^ The DPC database includes summarized inpatient information, such as recorded diagnoses of the disease that resulted in hospitalization, the cause of admission, comorbidities, and discharge status. The diseases were labeled according to the International Classification of Disease, 10th revision (ICD‐10) codes. The database also includes detailed information on the use of medical resources, diagnostic tests, surgical procedures, and prescribed and administered medications.

### 2.2 Patients

The study was conducted after obtaining approval from the Kyoto University Graduate School and Faculty of Medicine, Kyoto University Hospital Ethics committee in accordance with the guidelines on medical and epidemiological research (No. R135). Using the DPC database, we have identified inpatients of age <30 years at the time of receiving chemotherapy from July 2010 to March 2016. In Japan, there is no official age range definition for AYA.^12^ To capture many conditions, such as pregnancy, a range of 15‐39 years of age is often used. However, the aim of the study was to clarify the concordance with the antiemetic guidelines; therefore, we chose a definition of between 15 and 29 years of age for AYA.^12^ We collected the date of the first chemotherapy for each patient during the research period (Figure S1). The patients who were diagnosed with any cancer according to the ICD‐10 codes (C00‐D48) and who had received parenteral chemotherapeutic drugs with/without oral chemotherapeutic drugs, were included in the study.

### 2.3 Data collection

All data were collected from the DPC database; demographic characteristics including age, gender, diagnosis, chemotherapeutic drugs, antiemetic drugs, body weight, height, and first date of chemotherapy were collected. The chemotherapeutic drugs were classified according to the ASCO emetic risk category.^4^, ^5^ Although we can obtain the several guideline such as ASCO, MASCC/ESMO guideline in children and POGO (Pediatric Oncology Group of Ontario) guideline,^13^ we used ASCO for classification of the chemotherapy drugs that is suitable for the patients enroll of our study period. According to the ASCO guidelines, the emetic risk of cyclophosphamide and cytarabine depends on the dosage: a cyclophosphamide dose of ≥1500 mg/m^2^ has a high risk; cyclophosphamide <1500 mg/m^2^ and cytarabine >1000 mg/m^2^ doses have a moderate risk; and a cytarabine dose of ≤1000 mg/m^2^ has a low risk. We were required to predict the approximate dosage of cyclophosphamide and cytarabine to determine the emetic risk category. We used the Dubois formula to calculate the body surface area. In pediatric populations, the Mosteller formula is often used to calculate the body surface area, and there is a slight difference in the results obtained from these two formulas.^14^

### 2.4 Statistical analysis

We examined the prescription pattern of the prophylactic antiemetic drugs used (NK‐1RA, 5HT_3_RA, and dexamethasone) against the emetic risk of each intravenous chemotherapeutic drug. The percentage of patients who were administered prophylactic antiemetic drugs for chemotherapy was calculated, and the concordance with the 2006 and 2017 ASCO antiemetic guidelines was assessed (Figure S2). For the patients who had received chemotherapy multiple times, the data related to the first treatment were analyzed. The emetic risk was calculated based on the highest risk among the chemotherapeutic drugs prescribed on the same day. The antiemetic drugs that were prescribed on the same day as the chemotherapeutic drugs were considered as prophylactic.

The factors of discordance with the guidelines were determined by using logistic regression models. We classified the patients into the following seven age groups: 0‐2, 3‐4, 5‐9, 10‐14, 15‐19, 20‐24, and 25‐29 years of age. The independent variables were age group, gender, antiemetic risk category, and existing disease. The odds ratios (ORs) and 95% confidence intervals (CIs) for each variable were calculated. All the analyses were computed by using r statistical software (version 3.4.0) and a two‐sided significance level was fixed at 0.05.

## 3 RESULTS

### 3.1 Studied patients

In total, 21 106 patients who underwent chemotherapy were included. The patients were classified into the following seven age groups: 0‐2 (n = 2480), 3‐4 (n = 1417), 5‐9 (n = 2436), 10‐14 (n = 2528), 15‐19 (n = 3112), 20‐24 (n = 3513), and 25‐29 years of age (n = 5620). The median age was 16 years (range 0‐29 years) and the most common age category was 25‐29 years. The most frequent type of cancer was solid tumor (45.3%), and the most frequent emetic risk category was moderate (34.1%) (Table 1). The proportion of different types of cancer within each emetic risk category is shown in Table S1.

**Table 1:** Patient characteristics (N = 21 106)

Characteristic	n (%)
Gender	
Male	11 246 (53.4)
Female	9860 (46.7)
Age (y)	
Median (range)	16 (0‐29)
0‐2	2480 (11.8)
3‐4	1417 (6.7)
5‐9	2436 (11.5)
10‐14	2528 (12.0)
15‐19	3112 (14.7)
20‐24	3513 (16.6)
25‐29	5620 (26.6)
Disease	
Solid tumors	9562 (45.3)
Hematologic	9463 (44.8)
Brain tumor	2081 (9.9)
Received anticancer agents	
High emetic risk	6661 (31.6)
Moderate emetic risk	7188 (34.1)
Low emetic risk	5806 (27.5)
Minimal emetic risk	1451 (6.9)

The major cancer type among the patients who received moderate (42.2%), low (73.2%), or minimal (58.6%) risk category chemotherapy was hematologic malignancy, whereas the major cancer type among the patients who received chemotherapy in the high emetic risk category was solid tumor (72.4%) (Table S1). In addition, even when we analyzed only patients <18 years of age, the proportion of cancer type in each risk category was similar.

### 3.2 Prescription of prophylactic antiemetic drugs against chemotherapeutic drugs of different risk categories and concordance with the 2006 and 2017 ASCO antiemetic guidelines

With the high emetic risk chemotherapeutic drugs, the commonly used prophylactic antiemetic drugs were a combination of NK‐1RA, 5HT_3_RA, and steroids (51.1%) for patients ≥18 years of age, and 5HT_3_RA alone (48.2%) for patients <18 years of age. With the moderate and low emetic risk chemotherapeutic drugs, the most commonly used prophylactic antiemetic was 5HT_3_RA (43.0% and 52.0%, respectively). With the minimal emetic risk chemotherapeutic drugs, no prophylactic antiemetic was prescribed (51.6%) (Table 2).

**Table 2:** Details of prescription of each category of prophylactic antiemetic drugs and concordance with the ASCO antiemetic guidelines, 2006 and 2017

Antiemetic category (n)	Combination of agents	Recommendation of ASCO guidelines		% (95% CI)
		2006	2017	
Minimal (1451)	NK‐1RA + 5HT_3_RA + steroids	D	D	0.6 (0.2‐1.1)
	5HT_3_RA + steroids	D	D	3.2 (2.4‐4.3)
	NK‐1RA + 5HT_3_RA	D	D	3.0 (2.0‐4.0)
	NK‐1RA	D	D	0.3 (0.1‐0.8)
	5HT_3_RA	D	D	37.8 (35.0‐40.0)
	Steroids	D	D	3.4 (2.0‐4.0)
	None	Concordance	Concordance	51.6 (49.0‐54.2)
Low (5806)	NK‐1RA + 5HT_3_RA + steroids	D	D	1.5 (1.2‐1.8)
	5HT_3_RA + steroids	D	D	9.4 (8.7‐10.2)
	NK‐1RA + 5HT_3_RA	D	D	2.6 (2.2‐3.0)
	NK‐1RA + steroids	D	D	0.2 (0.1‐0.3)
	NK‐1RA	D	D	0.2 (0.1‐0.3)
	5HT_3_RA	D	Concordance	52.0 (50.7‐53.3)
	Steroids	Concordance	Concordance	5.9 (5.3‐6.6)
	None	D	D	28.3 (27.1‐29.4)
Moderate (7188)	NK‐1RA + 5HT3RA + steroids	D	D	14.0 (13.2‐14.8)
	NK‐1RA + steroids	D	D	0.1 (0.0‐0.20)
	NK‐1RA + 5HT_3_RA	D	D	7.3 (6.8‐8.0)
	Steroids + 5HT_3_RA	Concordance	Concordance	32.1 (31.0‐33.2)
	NK‐1RA	D	D	0.2 (0.1‐0.3)
	5HT_3_RA	D	D	43.0 (41.9‐44.2)
	Steroids	D	D	0.4 (0.30‐0.60)
	None	D	D	2.9 (2.5‐3.3)
High (≥18 y of age) (4130)	NK‐1RA + 5HT_3_RA + steroids	Concordance	Concordance	51.1 (49.5‐52.6)
	5HT_3_RA + steroids	D	D	20.1 (18.9‐21.4)
	NK‐1RA + 5HT_3_RA	D	D	9.7 (8.8‐10.7)
	NK‐1RA + steroids	D	D	0.3 (0.20‐0.50)
	NK‐1RA	D	D	0.2 (0.10‐0.30)
	5HT_3_RA	D	D	17.6 (16.5‐18.8)
	Steroids	D	D	0.3 (0.2‐0.50)
	None	D	D	0.7 (0.50‐1.00)
High (<18 y of age) (2531)	NK‐1RA + 5HT_3_RA + steroids	D	Concordance	21.5 (19.9‐23.2)
	5HT_3_RA + steroids	Concordance	D	18.2 (16.7‐19.8)
	NK‐1RA + 5HT_3_RA	D	D	10.2 (9.1‐11.5)
	NK‐1RA + steroids	D	D	0.2 (0.0‐0.40)
	NK‐1RA	D	D	0.2 (0.0‐0.40)
	5HT_3_RA	D	D	48.2 (46.2‐50.1)
	Steroids	D	D	0.2 (0.10‐0.50)
	None	D	D	1.3 (0.90‐1.90)

Abbreviations: NK‐1RA, neurokinin‐1 receptor antagonists; 5HT_3_RA, 5‐hydroxytryptamine_3_ receptor antagonists.

**Figure 1:**
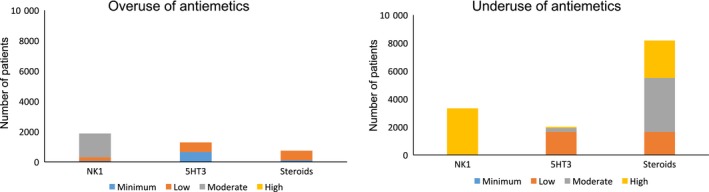
Overused or underused antiemetics. Analysis of the discordance for each antiemetic category

The concordance in each emetic risk category varied. Although up to 51.1% of the patients ≥18 years of age received the appropriate antiemetic drugs following the recommendation of the ASCO guidelines, only 21.5% patients <18 years of age received antiemetic drugs. The rate of concordance with the guidelines for moderate and minimal emetic risk chemotherapeutic drugs was 32.1% and 51.6%, respectively. The 2017 ASCO antiemetic guidelines are identical to the 2006 guidelines, except for the low emetic risk chemotherapeutic drugs. For the patients who had received low emetic risk chemotherapeutic drugs, the rate of concordance increased from 5.9% in the 2006 guidelines to 57.9% in the 2017 guidelines, reflecting the revision of the guidelines (Table 2). The factors of discordance were younger age, use of high and moderate emetic risk chemotherapeutic drugs, brain tumor, and hematological malignancy (Table 3). The reason for discordance was mainly the underuse of antiemetic drugs. In particular, steroids were less frequently prescribed to the patients who received low, moderate, and high emetic risk category chemotherapeutic drugs (Figure 1). The rate of overuse of NK1RA increased as age increased. In contrast, the rate of underuse of steroids increased as age decreased (Figure 1).

**Table 3:** Factors of concordance with the ASCO antiemetic guidelines, 2017

	Odds ratio	95% CI	*P* value
Gender	1.17	1.10‐1.24	<.001
Age (y)			
0‐2	0.53	0.48‐0.59	<.001
3‐4	0.73	0.64‐0.83	<.001
5‐9	0.80	0.72‐0.89	.001
10‐14	0.83	0.75‐0.91	.008
15‐19	0.80	0.73‐0.84	<.001
20‐24	0.96	0.88‐1.05	.598
25‐29	1		
Antiemetic category			
High emetic risk	0.51	0.46‐0.58	<.001
Moderate emetic risk	0.41	0.36‐0.46	<.001
Low emetic risk	1.31	1.16‐1.47	<.001
Minimal emetic risk	1		
Disease			
Hematologic malignancy	0.69	0.62‐0.77	<.001
Brain tumor	0.80	0.75‐0.85	<.001
Solid tumors	1		

## 4 DISCUSSION

This is the first study that demonstrates the concordance with the antiemetic guidelines in the pediatric population by using a large‐scale administrative database. Our study has four major findings.

First, the concordance with the antiemetic guidelines in the pediatric population was lower than that in the adults. Previous studies have shown that the rate of concordance with antiemetic guidelines was 70%‐90% in adults. We have found the concordance was approximately 20%‐60% in the pediatric population.

Second, this study clarified that the discordance was mainly caused by the underuse of antiemetics. We found that the prescription rate of steroids was low in all emetic risk categories. One reason for this was the clinicians' concerns about the adverse effects of steroids on the endocrine system and growth in the children. There is no consensus on the use of short‐term steroids for the treatment of emesis in children; hence, further research is needed. In cases of the overuse of antiemetics, we found that clinicians tended to prescribe NK1RA in the moderate risk category, or 5HT_3_RA in the low and minimal risk categories. NK1RA is more expensive than other antiemetics and there are risks of drug‐drug interaction; therefore, the appropriate use is recommended. For example, constipation is one of the known adverse effects of 5HT_3_RA, and vincristine and vinblastine, for which constipation is also an adverse effect, are representative minimal emetic risk drugs; therefore, the overuse of 5HT_3_RA should be avoided.

Third, the risk factors of discordance in the pediatric population are different from those in the adults. The risk factors of discordance with antiemetic guidelines in adults were hematological malignancy, older age, and the use of low emetic risk chemotherapeutic drugs.^8^, ^9^ In contrast, in the pediatric population, the risk factors were younger age, use of high emetic risk, moderate emetic risk chemotherapeutic drugs, brain tumor, and hematological malignancy. Although previous studies of adults showed that elderly patients were one of the risk factors for discordance, our study showed that younger patients were one of the risk factors. Namely, specific populations, such as elderly patients or younger children, might have additional issues that require consideration; comorbidities for elderly patients and concerns about the insomnia, indigestion/epigastric discomfort, agitation, increased appetite, weight gain, and acne for younger children.^15^ Many pediatric oncology protocols avoid dexamethasone due to concerns regarding potential interference with antitumor immunity,^16^, ^17^, ^18^ fungal infection^19^ and distribution of chemotherapy across the blood brain barrier.^20^ These issues may influence the decision to prescribe antiemetic for these populations. Indeed, the POGO guidelines^10^, ^21^ take the use of steroids into consideration, and there is a slight difference between POGO and ASCO guidelines, mainly in the high emetic risk category, although the content of these guidelines is similar (Table S2). In addition, steroids are prescribed for hematologic malignancy with therapeutic intent, and clinicians prioritize the therapeutic administration over adherence to antiemetic guidelines. Alternatively, in the case of brain tumor, steroids can be prescribed for symptom management.

Fourth, the rate of concordance was improved by the updated 2017 ASCO antiemetic guidelines. The 2017 ASCO antiemetic guidelines recommend the use of 5HT_3_RA along with low emetic risk chemotherapeutic drugs. As a result, the rate of concordance with the 2017 ASCO antiemetic guidelines for low emetic risk chemotherapeutic drugs was increased from 5.9% (in 2006) to 57.9%. We found that the clinicians were already prescribing 5HT_3_RA‐containing antiemetic regimens to the patients who had received low emetic risk chemotherapeutic drugs before the 2006 guidelines were updated.

Recently, NK‐1RA has been recommended along with the high emetic risk chemotherapeutic drug regimens in the pediatric population. The rate of concordance with the guidelines with high emetic risk chemotherapeutic drugs was low in our study. One possible reason for this was that the use of NK‐1RA for patients under 12 years of age has only been permitted in Japan only from April 2016.

A recent Canadian study of 200 patients, evaluating the rate of prescription concordance to the CINV guidelines showed that only 29% of the prescriptions containing high and moderate emetic risk chemotherapeutic drugs were concordant with the guidelines, resulting in poor control of CINV.^22^ Therefore, our results that indicated the low rate of concordance with the antiemetic guidelines in the pediatric population may suggest that the quality of life in the children undergoing treatment with cancer chemotherapeutic drugs be affected.

Our study has several limitations. First, our study considered only the chemotherapeutic drug‐induced acute emesis and did not consider delayed emesis. The guidelines mainly recommend the use of antiemetic drugs during acute emesis in the pediatric population. Further studies on the use of prophylactic drugs to prevent delayed emesis are needed. Second, the DPC database did not provide patients outcomes, such as vomiting rates, medical history, and laboratory results of patients. Third, the assumption that all centers used ASCO as their source guidelines for antiemetic prescribing was made. We need to interview the medical staff in the centers that treat pediatric and AYA patients with cancer to determine which guidelines were used in daily practice.

In conclusion, our study has identified a substantial scope to improve the antiemetic practice and mitigating the risk factors of discordance with the antiemetic guidelines in the pediatric, adolescent, and young adult patients. These identified risk factors were different from those in the adult population. Further studies to evaluate the causes of this discordance are warranted.
