# Supplementary figures and images for: Analysis of concordance with antiemetic guidelines in pediatric, adolescent, and young adult patients with cancer using a large‐scale administrative database
Source: Cancer Med. 2019 Aug 30;8(14):6243–9. doi: 10.1002/cam4.2486 (PMC6797697; doi:10.1002/cam4.2486)

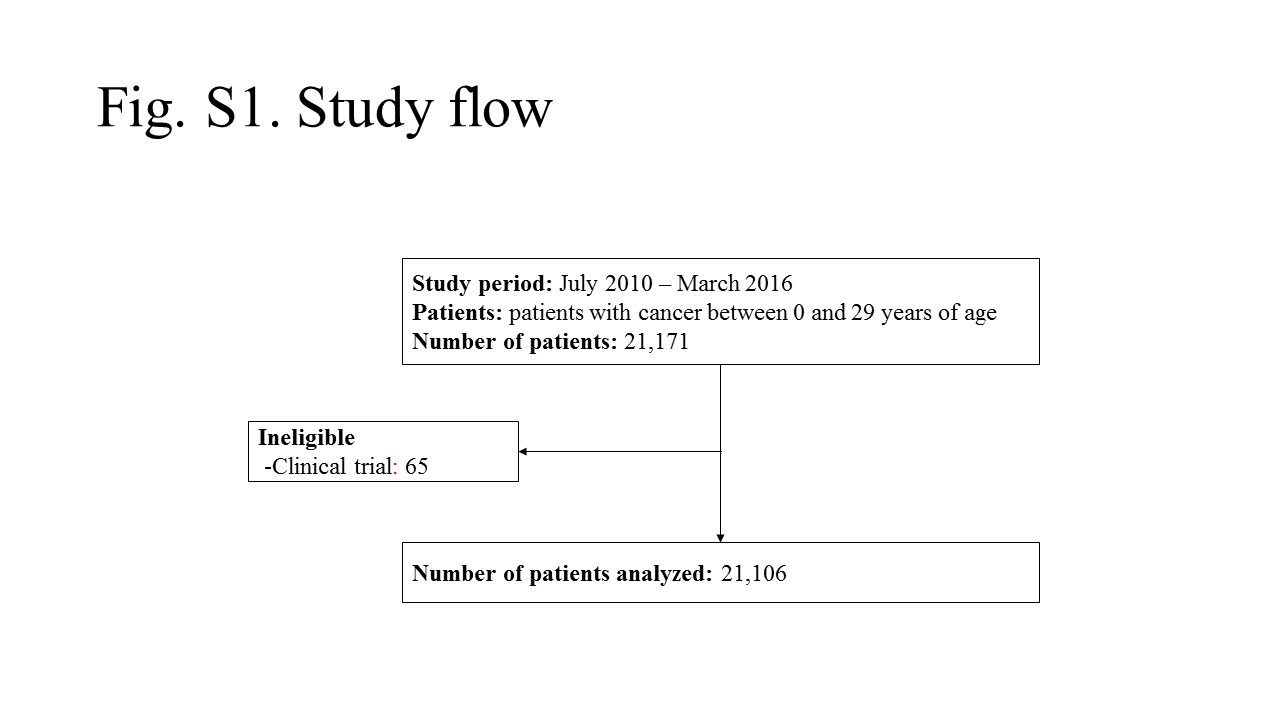

Supplement: Supplementary file 1 [file CAM4-8-6243-s001.tif]

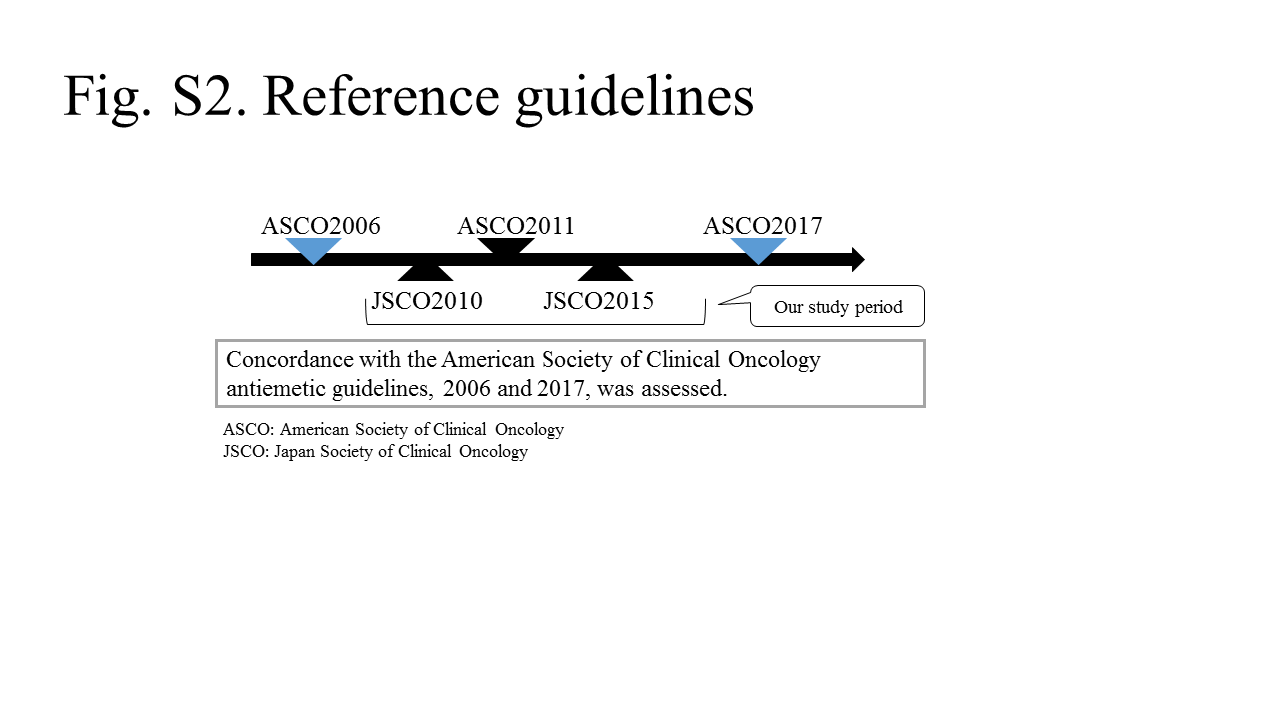

Supplement: Supplementary file 2 [file CAM4-8-6243-s002.tif]
