# Supplementary material for: Analysis of concordance with antiemetic guidelines in pediatric, adolescent, and young adult patients with cancer using a large‐scale administrative database
Source: Cancer Med. 2019 Aug 30;8(14):6243–9. doi: 10.1002/cam4.2486 (PMC6797697; doi:10.1002/cam4.2486)
Supplement: Supplementary file 3 [file CAM4-8-6243-s003.docx]

|  | Category of emetic risk % (95% CI) | | | | | | | |
| --- | --- | --- | --- | --- | --- | --- | --- | --- |
|  | High emetic risk | | Moderate emetic risk | | Low emetic risk | | Minimum emetic risk | |
|  | All age  (n = 6,661) | <18 yo  (n =2,531 ) | All age  (n = 7,188) | <18 yo  (n =3,338) | All age  (n = 5,806) | <18 yo  (n = 3,961) | All age  (n = 1,451) | <18 yo  (n = 909) |
| Disease |  |  |  |  |  |  |  |  |
| -Hematologic | 1,333 (20.0) | 186 ( 7.3) | 3,030 (42.2) | 1381 (41.4) | 4,249 (73.2) | 3413 (86.1) | 851 (58.6) | 407 (44.8) |
| -Solid tumors | 4,825 (72.4) | 1948 (77.0) | 2,998 (41.7) | 1016 (30.4) | 1,281 (22.1) | 389 ( 9.8) | 458 (31.6) | 376 (41.4) |
| -Brain tumor | 503 ( 7.6) | 397 (15.7) | 1,160 (16.1) | 941 (28.2) | 276 ( 4.8) | 160 ( 4.0) | 142 ( 9.8) | 126 (13.9) |

Table S1 Rating of prescription of an emetic risk category for each cancer

n (%)
