# Supplementary material for: Analysis of concordance with antiemetic guidelines in pediatric, adolescent, and young adult patients with cancer using a large‐scale administrative database
Source: Cancer Med. 2019 Aug 30;8(14):6243–9. doi: 10.1002/cam4.2486 (PMC6797697; doi:10.1002/cam4.2486)
Supplement: Supplementary file 4 [file CAM4-8-6243-s004.docx]

Table S2 Details of prescription of each category of prophylactic antiemetic drugs for pediatrics and concordance with the antiemetic guidelines, ASCO 2017 and POGO 2017.

|  |  | Recommendation of guidelines | |  |
| --- | --- | --- | --- | --- |
| Antiemetic category (n) | Combination of agents | POGO 2017 | ASCO 2017 | % (95% CI) |
| Minimal (902) | NK-1RA + 5HT_3_RA + steroids | D | D | 0.1 (0.0 -0.6) |
|  | 5HT_3_RA + steroids | D | D | 3.9 (2.7 – 5.3) |
|  | NK-1RA + 5HT_3_RA | D | D | 2.2 (1.3 – 3.4) |
|  | NK-1RA | D | D | 0.2 (0.0 - 0.8) |
|  | 5HT_3_RA | D | D | **49.5 (46.2 – 52.8)** |
|  | Steroids | D | D | 3.2 (2.1 – 4.5) |
|  | None | Concordance | Concordance | 40.9 (37.7 - 44.2) |
| Low (3,962) | NK-1RA + 5HT_3_RA + steroids | D | D | 0.8 (0.6 – 1.1) |
|  | 5HT_3_RA + steroids | D | D | 6.9 (6.1 – 7.7) |
|  | NK-1RA + 5HT_3_RA | D | D | 2.0 (1.6 – 2.5) |
|  | NK-1RA | D | D | 0.1 (0.0 – 0.3) |
|  | 5HT_3_RA | Concordance | Concordance | **66.0 (64.5 – 67.5)** |
|  | Steroids | D | Concordance | 1.7 (1.3 – 2.1) |
|  | None | D | D | 22.4 (21.1 – 23.8) |
| Moderate (3,338) | NK-1RA + 5HT3RA + steroids | D | D | 6.1 (5.4 – 7.0) |
|  | NK-1RA + steroids | D | D | 0.0 (0.0 – 0.20) |
|  | NK-1RA + 5HT_3_RA | D | D | 6.2 (5.4 – 7.1) |
|  | Steroids + 5HT_3_RA | Concordance | Concordance | 26.9 (25.4 – 28.4) |
|  | NK-1RA | D | D | 0.3 (0.1 – 0.5) |
|  | 5HT_3_RA | D | D | **58.1 (56.4 – 59.7)** |
|  | steroids | D | D | 0.5 (0.30 – 0.80) |
|  | None | D | D | 1.9 (1.5 – 2.5) |
| High (2,531) | NK-1RA + 5HT_3_RA + steroids | Concordance | Concordance | 21.5 (19.9-23.2) |
|  | 5HT_3_RA + steroids | Concordance* | D | 18.2 (16.7- 19.8) |
|  | NK-1RA + 5HT_3_RA | Concordance** | D | 10.2 (9.1 – 11.5) |
|  | NK-1RA + steroids | D | D | 0.2 (0.0 – 0.40) |
|  | NK-1RA | D | D | 0.2 (0 .0– 0.40) |
|  | 5HT_3_RA | D | D | 48.2 (46.2 – 50.1) |
|  | steroids | D | D | 0.2 (0.10 - 0.50) |
|  | None | D | D | 1.3 (0.90 – 1.90) |

ASCO: American Society for Clinical Oncology

POGO: Pediatric Oncology Group of Ontario

NK-1RA: neurokinin-1 receptor antagonists, 5HT_3_RA: 5-hydroxytryptamine_3_ receptor antagonists

* It is known or suspected to interact with aprepitant.

** It is not known or suspected to interact with aprepitant, and who cannot receive dexamethasone for CINV.
